# Natural Language Processing Insight into LGBTQ+ Youth Mental Health During the COVID-19 Pandemic: Longitudinal Content Analysis of Anxiety-Provoking Topics and Trends in Emotion in LGBTeens Microcommunity Subreddit

**DOI:** 10.2196/29029

**Published:** 2021-08-17

**Authors:** Hannah R Stevens, Irena Acic, Sofia Rhea

**Affiliations:** 1 Department of Communication University of California, Davis Davis, CA United States

**Keywords:** COVID-19, natural language processing, LGBTQ+, mental health, anxiety, emotion, coronavirus, outbreak

## Abstract

**Background:**

Widespread fear surrounding COVID-19, coupled with physical and social distancing orders, has caused severe adverse mental health outcomes. Little is known, however, about how the COVID-19 crisis has impacted LGBTQ+ youth, who disproportionately experienced a high rate of adverse mental health outcomes before the COVID-19 pandemic.

**Objective:**

We aimed to address this knowledge gap by harnessing natural language processing methodologies to investigate the evolution of conversation topics in the most popular subreddit for LGBTQ+ youth.

**Methods:**

We generated a data set of all r/LGBTeens subreddit posts (n=39,389) between January 1, 2020 and February 1, 2021 and analyzed meaningful trends in anxiety, anger, and sadness in the posts. Because the distribution of anxiety before widespread social distancing orders was meaningfully different from the distribution after (*P*<.001), we employed latent Dirichlet allocation to examine topics that provoked this shift in anxiety.

**Results:**

We did not find any differences in LGBTQ+ youth anger and sadness before and after government-mandated social distancing; however, anxiety increased significantly (*P*<.001). Further analysis revealed a list of 10 anxiety-provoking topics discussed during the pandemic: attraction to a friend, coming out, coming out to family, discrimination, education, exploring sexuality, gender pronouns, love and relationship advice, starting a new relationship, and struggling with mental health.

**Conclusions:**

During the COVID-19 pandemic, LGBTQ+ teens increased their reliance on anonymous discussion forums when discussing anxiety-provoking topics. LGBTQ+ teens likely perceived anonymous forums as safe spaces for discussing lifestyle stressors during COVID-19 disruptions (eg, school closures). The list of prevalent anxiety-provoking topics in LGBTQ+ teens’ anonymous discussions can inform future mental health interventions in LGBTQ+ youth.

## Introduction

The COVID-19 pandemic has dramatically affected both physical and mental health worldwide. As of February 1, 2021, the novel coronavirus infected over 100 million people in the United States and has killed over 2.5 million people globally [1]. Widespread fear about COVID-19, coupled with physical and social distancing orders, has caused severe adverse mental health outcomes and interpersonal relationship turmoil [2,3]. Before the pandemic, 1 in 10 US adults had symptoms of anxiety or depressive disorder. By January 2021, this figure had increased to 4 in 10 adults [4].

This sharp mental health decline may be different for LGBTQ+ youth, who disproportionately experienced a high rate of adverse mental health outcomes before the COVID-19 pandemic due to prejudice, victimization, and unaccepting communities [5-7]. As of 2017, rates of suicidal ideation were 4 times greater in LGBTQ+ youth than those for their heterosexual, cisgender peers [8]. Amid stay-at-home orders, school closures, rollbacks of LGBTQ+ nondiscrimination protections, and the stresses of being home in potentially unsupportive environments, LGBTQ+ youth are even more vulnerable to mental health struggles during the COVID-19 crisis [5,9].

LGBTQ+ youth report that cost and parental consent are barriers to accessing mental health resources, and the inability to access confidential school counseling during COVID-19 school closures magnifies these obstacles [10,11]. Anonymous, confidential, and free online support groups are safe resources for LGBTQ+ youth during widespread school closures. LGBTQ+ youth’s use of web-based platforms for support may be reinforced by the popularity of web-based platforms among younger generations as a means to create and maintain connections [5]. Decreased access to mental health resources and counseling, paired with a lack of family support, make anonymous discussion forums practical outlets for LGBTQ+ youth.

Questioning one’s sexuality is a normal developmental aspect of adolescence [12]. Pubescent adolescents may experience same-sex attraction that causes them to question their sexual orientation [12]. Over time, adolescents become more certain of their sexuality and develop different sexual orientations and gender identities. As a result, individuals, who at one point disclose they are straight and cisgender, may later identify as LGBTQ+.

As individuals begin to realize their sexual orientation, they may choose to self-disclose their identity. Scholars conceptualize self-disclosure of sexual and gender identity as a dimension of the coming-out process that is closely linked to self-esteem, emotional distress, and well-being [13,14]. Yet LGBTQ+ youth may be hesitant to disclose their gender and sexual identities for fear of stigmatization, which spans across a variety of contexts, such as health care and education [15-17]. LGBTQ+ youth strategically tailor their identity disclosure to distinct social contexts to manage their stigmatized identities [18,19].

Research shows that LGBTQ+ youth resort to computer-mediated communication to explore their identities and find community [20]. For example, teens who do not disclose their sexuality to their classmates may feel comfortable revealing their sexuality to anonymous support forums for LGBTQ+ youth. Furthermore, the isolating nature of the COVID-19 pandemic makes anonymous discussion forums especially viable outlets for naturalistic studies of LGBTQ+ youth mental health. In online anonymous discussion forums, users can seek support and forge connections, which they might access otherwise, during isolation orders and school closures.

Additionally, because gender and sexual minorities are highly stigmatized, LGBTQ+ youth may not be comfortable disclosing their gender or sexual orientation to researchers as part of formal surveys and experiments [12]. Because it allows anonymity, the LGBTeens subreddit is a microcommunity that is well-suited to the investigation of an otherwise hard-to-reach population; it is a popular microcommunity that focuses on LGBTQ+ issues and youth. While in some subreddit spaces, users’ stigmatized identities might be faced with incivility [21], the norms of the LGBTQ+ microcommunity dictate that it is a safe space for LGBTQ+ youth to seek support in a space that validates their stigmatized identities.

In summary, the COVID-19 crisis has caused a concurrent mental health crisis. LGBTQ+ youth are especially vulnerable to adverse mental health outcomes, and online anonymous support forums are a uniquely accessible resource for LGBTQ+ youth to disclose their identities during the pandemic. LGBTQ+ self-disclosure is helpful for LGBTQ+ youth’s mental health [13,14], yet research is needed to investigate how this vulnerable population manages their stigmatized identities while coping with the unique challenges of the pandemic and widespread social unrest [22].

However, at the time of this study, no longitudinal studies have investigated how discussions of the themes and sentiment of LGBTQ+ youth support forums unfold. We aimed to address this knowledge gap. We raised the following question: What patterns of emotions emerge from longitudinal analyses of LGBTQ+ youth conversation during the COVID-19 crisis?

Given that LGBTQ+ youth were disproportionately vulnerable to adverse mental health outcomes before the pandemic [5-7], we were interested in understanding how LGBTQ+ youth were impacted relative to the wider population of youth affected by lifestyle stressors during the COVID-19 crisis. Furthermore, research has revealed that individuals of all ages experienced interpersonal relationship turmoil during the COVID-19 crisis [3]; thus, we were interested in understanding whether this pattern was specific to teenagers or similar to the trajectory of all interpersonal relationships experiencing relationship turmoil related to the COVID-19 crisis—regardless of age. Accordingly, we posed the following question: How does the trajectory of patterns of emotions emerging from LGBTQ+ youth conversation compare to the patterns of emotions emerging from the wider population of youth as well as those emerging from any interpersonal relationships during the COVID-19 crisis?

In addition to being suitable for naturalistic investigations of emotion over time, online communities can illuminate which topics contribute to meaningful emotional trends. Knowing which topics are emotionally distressing to LGBTQ+ individuals is a requisite precursor to informing LGBTQ+ youth mental health interventions, yet at the time of this study, none had investigated themes related to LGBTQ+ youth online forums during the COVID-19 pandemic. To address this gap in the literature, we raised the following question: What conversation topics manifest from meaningful emotional trends?

## Methods

### Data Set

The pushshift (version 4.1) Python (version 3.9.0) package was used to extract all public posts made between January 1, 2020 and January 31, 2021 from the r/LGBTeens subreddit (n=38,389 posts). We chose this online community because of its popularity as a community for LGBTQ+ youth and its specific focus on teens. Because we aimed to assess how users’ textual expressions manifested amid global events, not in response to others’ posts, comments were excluded from the data set. Although the anonymity of Reddit prevented us from accessing demographic information about the r/LGBTeens community, Reddit users live predominantly in the United States (49.3%) [23]. Notably, given the integral role of the United States in promoting LGBTQ+ rights in foreign policy, even LGBTQ+ youth outside the United States are impacted by the erosion of US LGBTQ+ advocacy [22].

To understand whether r/LGBTeens emotional patterns were specific to LGBTQ+ teenagers, we compared the trajectory of emotional tone in r/LGBTeens posts with those in 2 other subreddit microcommunities. After a review of relevant subreddits, we determined that r/Teenagers was the largest subreddit community, with n=1,364,980 posts, tailored toward a wide population of teens. To investigate LGBTeens post sentiment relative to widespread interpersonal relationship turmoil during the COVID-19 crisis [3], we compared the emotional tones of r/LGBTeens and r/Teenagers posts to that in the average of r/Relationships posts over time; r/Relationships was the largest subreddit dedicated to posts about interpersonal relationships (n=193,282).

This study only used information that could be accessed freely by the public. This study did not include any personally identifiable information. The institutional review board recognized that analysis of publicly available data does not constitute research on human participants. Thus, ethical review approval was not required for this study.

### Trend Analysis

To track negative emotions over time, we analyzed aggregate post sentiment using the Linguistic Inquiry and Word Count program (LIWC) [24]. LIWC is a computerized coding tool that analyzes psychological processes (eg, positive and negative emotions) in texts by calculating the percentage of words in prevalidated lexicons relative to all words in a text. For example, we might find that 22 of 230 (9.56%) words in a post were words related to anxiety (eg, “scared” or “stressed”), and the program would assign that particular post an anxiety score of 9.56.

We focused on levels of anger, sadness, and anxiety present in posts because these psychological processes are symptoms of COVID-19–induced mental health challenges. For example, COVID-19 health threats and uncertainty may trigger feelings of anxiety [25-27]. Similarly, the COVID-19 pandemic has presented persistent stressful stimuli that some individuals may react to with anger [28-30]. Current research has demonstrated a positive correlation between the perceived threat of COVID-19 and moods of anger and hostility [31].

Likewise, the loss of loved ones, feelings of isolation, and routine disruptions associated with the rapidly changing COVID-19 pandemic may trigger feelings of sadness. For example, a recent study [32] showed a link between increased perceived loneliness and depression symptoms. Similarly, a longitudinal study [33], conducted with children in the United Kingdom, demonstrated a substantial increase in childhood depression symptoms compared to childhood depression symptoms before lockdowns began. As anonymous support forums serve as space for individuals to build community resilience in times of crisis [34], we analyzed the overall emotional tone of posts (positive vs negative) using LIWC to understand the potential positive influences of events (eg, the US presidential election of Joe Biden, a supporter of LGBTQ+ rights) on LGBTQ+ youth mental health [35]. Furthermore, we holistically investigated the relative emotional valence of texts over time. To investigate if patterns of emotion were specific to members of the LGBTQ+ youth community, or general to the population, we compared the trajectory of LIWC emotional tone scores of r/LGBTeens posts to those of 2 other subreddit spaces.

### Lifestyle Stressors

We explored differences in the trajectory of emotions displayed in posts by visualizing trends. In addition, we recorded salient events during the crisis to examine how they may have affected the changing patterns of COVID-related user responses and associated emotions.

We marked 10 major events in the course of the COVID-19 crisis. Events were selected if they considerably disrupted LBGTQ+ youth lifestyles (eg, widespread school closures) [36,37] or if they affected the rights of the wider LBGTQ+ community [38-41]. According to the Centers for Disease Control and Prevention, the first US coronavirus cases emerged on January 21, 2020 [36]. In mid-February, many school campuses were closed temporarily to assess the severity of the virus [37]. Permanent campus closure then followed on March 16, 2020 [37]. By May 7, 2020, many schools announced that they were extending web-based learning for the remainder of 2020 [37]. School closures and shifts toward web-based learning represented a turbulent time for many adolescents during the pandemic. In addition, Black Lives Matter protests in the US peaked in June 2020, which garnered support from LGBTQ+ Pride celebrations around the world [38-40]. Black Lives Matter and Pride were followed by widespread political unrest for LGBTQ+ populations, including a change by the Trump administration that eliminated nondiscrimination protections for LGBTQ+ health care on August 8, 2020, as well as the Supreme Court nomination of Amy Coney Barrett [41]. We marked both events, as well as the election of President Biden, an advocate of LGBTQ+ populations when compared to President Trump [35,41]. Finally, we marked the January 6 Capitol riots, which threatened advocates of LGBTQ+ rights as well as President Biden’s inauguration [35].

### Topic Analysis

We extrapolated meaningful conversation topics related to trends in anxiety, anger, sadness, as well as, overall emotional tone. We conducted 2-tailed independent sample *t* tests to determine whether the mean levels of anxiety, sadness, and anger were significantly different before and after March 21, 2020, when COVID-19 social distancing orders were enacted. To check for homogeneity of variance, we ran Levene tests for each of the 3 variables. Although results revealed the variance of sadness (*F*_1,39352_=0.11, *P*=.74) and anger (*t*_39352_=−0.57, *P*=.57) met the assumption, the variance of anxiety (*F*_1,39352_=37.99, *P*<.001) violated the assumption. We ran a 2-tailed Mann-Whitney 2-sample rank-sum test to supplement the results.

The results of the 2-tailed independent t tests suggested differences in the mean of anger (*t*_39352_=−0.57, *P*=.57) and sadness (*t*_39352_=−0.34, *P*=.74) were not meaningfully different before and during the widespread social distancing orders. However, results revealed the mean of anxiety was significantly higher during social distancing measures (*t*_17672.02_=−7.94, *P*<.001). The distribution of anxiety before widespread social distancing orders was meaningfully different from the distribution after (*U*=125657033, *z*=−6.20, *P*<.001). To supplement the conclusion that anxiety levels decreased as a function of lifestyle stressors related to the COVID-19 crisis, we plotted the anxiety levels of posts with anxiety-related topics from January 1, 2015 through January 31, 2021. We found an unprecedented upsurge between January 1, 2020 and January 1, 2021 (Multimedia Appendix 1). Next, we employed latent Dirichlet allocation (LDA) to examine topics provoking this shift in anxiety.

LDA topic modeling is a bag-of-words machine learning algorithm that extrapolates meaningful topics from a large body of texts, in this case, subreddit posts [42]. Notably, we excluded posts with 0 LIWC anxiety from the LDA model. In line with the LDA procedures and guidelines outlined by prior researchers, we preprocessed the textual data and selected the optimal number of topics, and then human coders labeled those topics [43].

### Text Processing

To generate a bag of words for the LDA model, we preprocessed the texts by tokenizing the text, removing stop words, lemmatizing the text, and generating a document term matrix. Tokenization separates sentences into bags of unordered words by removing all punctuation and making words lowercase. Given our relatively small sample size (n=7882 texts), lemmatization was necessary to reduce model noise. Lemmatizing texts removes prefixes and suffixes by transforming all words to their base lemma (eg, we converted “vaccinated” and “vaccinating” to “vaccine”). Only nouns were retained through the process of lemmatization because other parts of speech were not meaningful to our topics.

### Model Selection

We set β to learn the asymmetric prior from the data [44]. Because multiple topics may have appeared in a single subreddit post, we set α=.9 to reflect the nuanced distribution of topics per text. To identify the optimal number (*k*) of topics for the model (Multimedia Appendix 2), we used the perplexity metric (the normalized log-likelihood of the model finding a previously unseen term), in which lower perplexity values suggest greater model accuracy. To keep the model parsimonious, we compared the perplexity values of models with *k*=1 to 30 topics and selected the 11 topic model because it yielded the lowest *k* value relative to the lowest perplexity value (−7.68).

### Topic Labeling

Topics defined by the model require human labeling. LDA generates a list of the most relevant 30 terms, along with each term’s β value (ie, their relative contribution to that topic) (Multimedia Appendix 3). The algorithm also identifies posts that belong primarily to a single topic. Two coders individually inspected the top 10 most relevant posts and the 30 terms with the highest β values, and then used these terms to label each topic. Human coders deemed 1 topic incoherent (Multimedia Appendix 4).

A third human coder validated the labels by reviewing the top 10 most relevant posts for each topic and confirming the human coder–assigned labels are present in those posts. The third human coder confirmed that the incoherent topic was indeed incoherent (Multimedia Appendix 5).

## Results

### Patterns of LGBTQ+ Teen Negative Emotion During the COVID-19 Crisis

**Figure 1:**
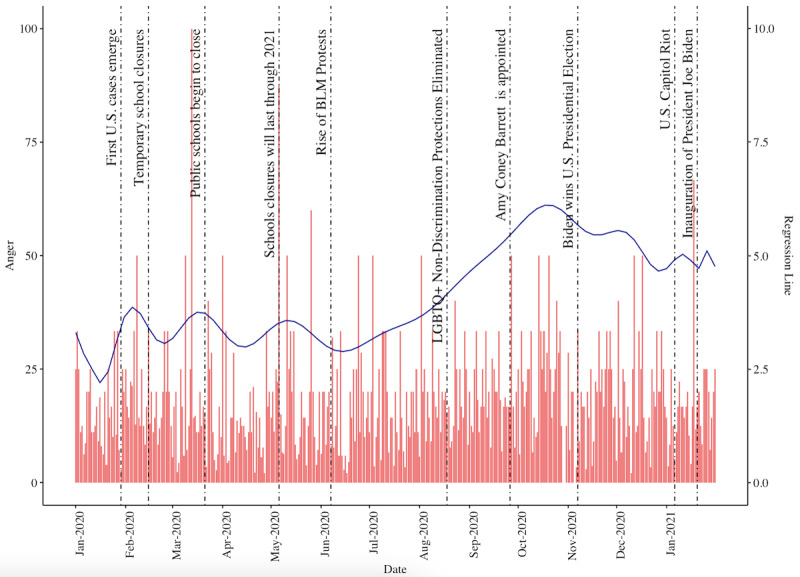
Histogram of the percentage of angry sentiment observed in r/LGBTeens posts over time, juxtaposed with a solid blue polynomial regression line showing average anger levels observed in angry r/LGBTeens posts over time. LGBTQ+: Lesbian, Gay, Bisexual, Transgender, Queer/Questioning, and Others.

We quantified the proportion of angry sentiment observed in r/LGBTeens posts relative to each post’s total number of words. Of the 39,389 posts in the data set, 17.67% (6961) were classified as containing anger. The mean percentage of words denoting anger relative to the total number of words in an anger-flagged post was 4.72% (SD 8.81%). There was an upsurge of anger following widespread school closures in the United States, and a second upsurge when many schools announced closures would last through the end of 2020 (Figure 1). Anger increased significantly again after the rise of the Black Lives Matter protests. More specifically, anger levels almost doubled between the Black Lives Matter protests on June 7, 2020 and when Biden won the US presidential election on November 7, 2020 [45,46]. The upsurge of anger may have been a result of frustration surrounding the Trump administration’s stance on LGBTQ+ and racial injustice [47,48], which resulted in policies such as the elimination of nondiscrimination protections for LGBTQ+ health care on August 8, 2020 [41].

**Figure 2:**
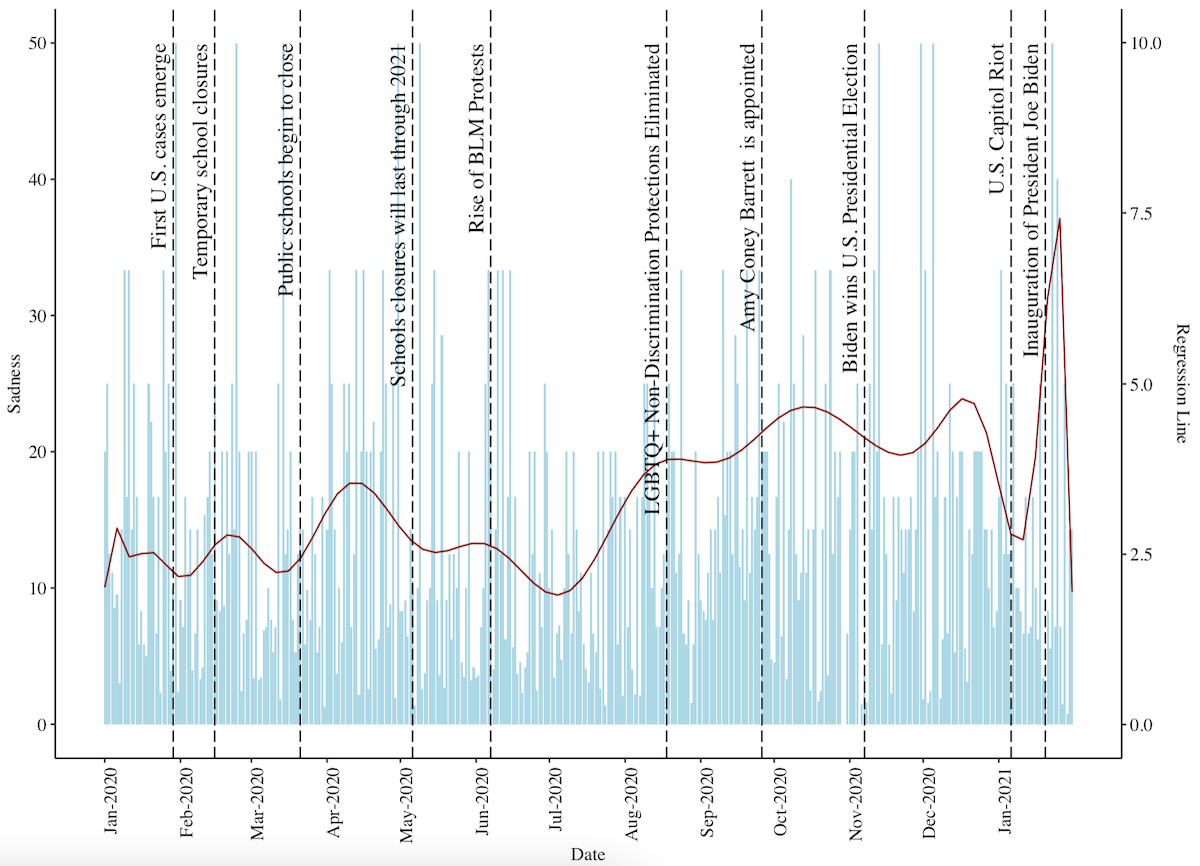
Histogram of the percentage of sad sentiment observed in r/LGBTeens posts over time, juxtaposed with a solid red polynomial regression line showing average sadness levels observed in sad r/LGBTeens posts over time. BLM: Black Lives Matter; LGBTQ+: Lesbian, Gay, Bisexual, Transgender, Queer/Questioning, and Others.

We also quantified the proportion of sad sentiment observed in r/LGBTeens posts relative to the total number of words in each post (Figure 2). Sadness appeared in 14.4% (5687/39,389) of the posts in the data set (mean 3.82, SD 8.47). There was an upsurge of posts with high sad sentiment when the Trump administration enacted a change that eliminated nondiscrimination protections for LGBTQ+ health care on August 8, 2020 [41]. We found another increase in sadness when Amy Coney Barrett was appointed to the US Supreme Court [41]. However, following the election of President Biden—an advocate of LGBTQ+ rights when compared to President Trump [35,41]—there was a decrease in sadness until the Capitol riots on January 6, when sadness levels sharply increased. Sadness levels decreased again after President Biden’s first week in office, perhaps because of positive feelings about President Biden’s pro-LGBTQ+ policies [35].

**Figure 3:**
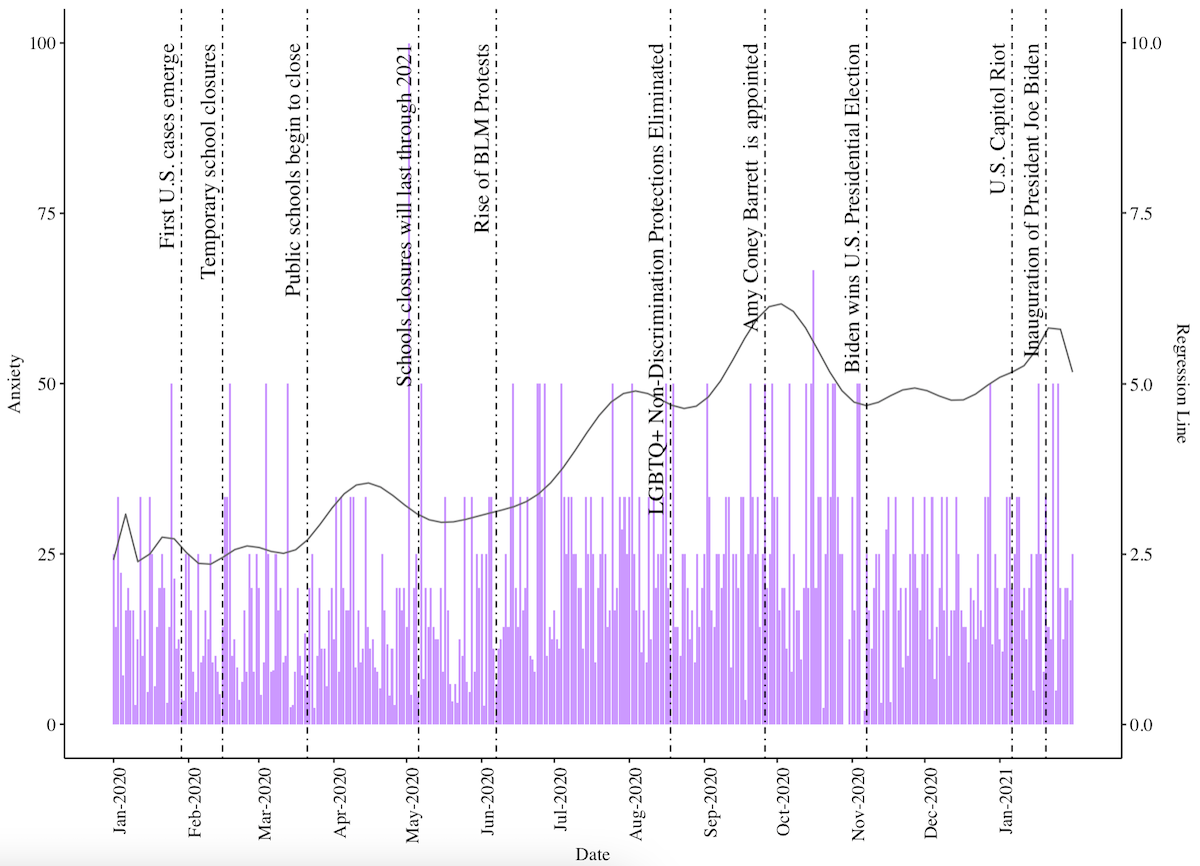
Histogram of the percentage of anxiety sentiment observed in r/LGBTeens posts over time, juxtaposed with a solid grey polynomial regression line showing average anxiety levels observed in anxious r/LGBTeens posts. BLM: Black Lives Matter; LGBTQ+: Lesbian, Gay, Bisexual, Transgender, Queer/Questioning, and Others.

Of the 39,389 posts in the data set, 20.01% (7881) were classified as containing anxiety (anxiety level: mean 4.97, SD 10.43). Although all 3 negative emotions analyzed in r/LGBTeens posts increased over time, anxiety trended upward the most sharply. The histograms reveal a sharp spike in anxiety, sadness, and anger in the first week of May 2020, which may reflect emotional distress resulting from US schools closing for the remainder of the school year (Figure 3). Another upsurge in anxiety occurred in late October, potentially a result of concern surrounding the future of LGBTQ+ constitutional rights following the Supreme Court appointment of Amy Coney Barrett [41]. This spike in anxiety flattened after President Biden won the US presidential election but spiked again on January 6, 2021, which was the day of the US Capitol riot [49].

To supplement the conclusion that the mental health of r/LGBTeens community members had been adversely impacted by the COVID-19 crisis and concurrent social and political unrest, we measured the overall emotional tone of each post from the expanded timeline of January 1, 2015 through January 31, 2021. Emotional tone is measured by LIWC as the valence of texts (ie, whether a text is positively valenced or negatively valenced) [24].

**Figure 4:**
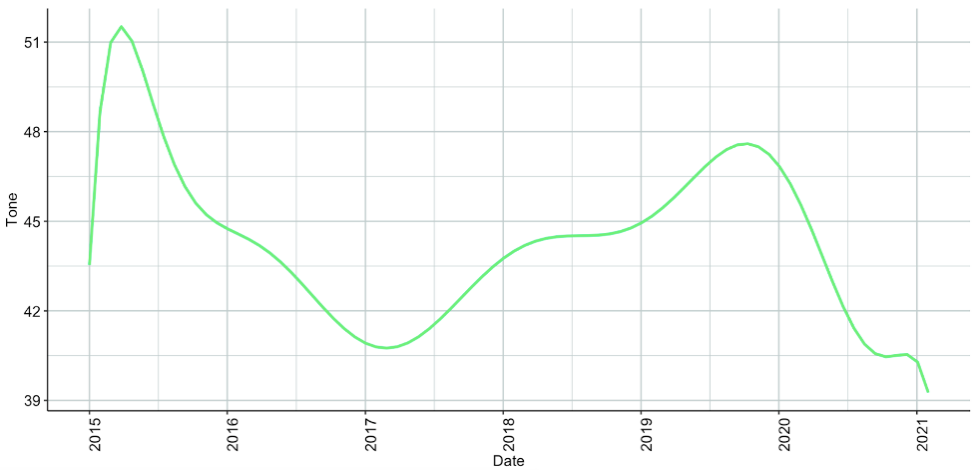
Green polynomial regression line representing mean emotional tone of r/LGBTeens posts from January 1, 2015 through January 31, 2021. We note a sharp decrease in the emotional tone of posts during 2020.

Of all posts encompassed in the 5-year period (n=123,440), the average post valence was negative (mean 44.33, SD 35.11, SEM 0.10, minimum 1.00, maximum 99.00, skewness 0.53, kurtosis −1.25). We found that posts became more negatively valenced throughout 2020 (Figure 4).

**Figure 5:**
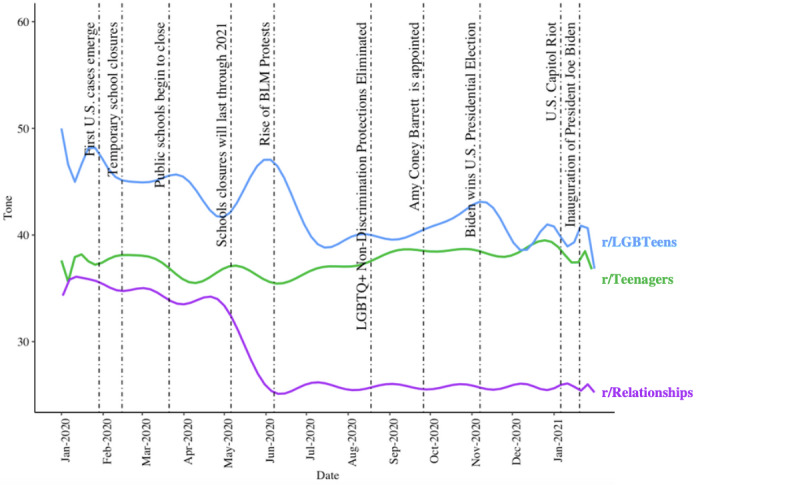
Polynomial regression line representing the average post emotional tone (values of 100 represent maximally positive emotional tone; values below 50 represent more negatively valenced tone) of 3 subreddit communities (r/LGBTeens, r/Teenagers, and r/Relationships). BLM: Black Lives Matter; LGBTQ+: Lesbian, Gay, Bisexual, Transgender, Queer/Questioning, and Others.

We compared the emotional tone of r/LGBTeens posts from January 1, 2020 to January 31, 2021 (n=38,389 posts) to the emotional tone of r/Teenagers (n=1,364,980) and r/Relationships (n=193,282) posts from the same time period (Figure 5). The mean emotional sentiment of r/LGBTeens posts was 42.34 (SD 34.68, SEM 0.18, minimum 1.00, maximum 99.00). Overall post valence in the r/Teenagers subreddit (n=1,364,980 posts) was negative (mean 37.4, SD 32.95, SEM 0.03, minimum 1.00, maximum 99.00, skewness 0.94, kurtosis −0.52). Overall post valence in the r/Relationships subreddit (n=193,282) was negative (mean 28.38, SD 15.57, SEM 0.04, minimum 1.00, maximum 99.00, skewness 2.27, kurtosis 6.84).

A 2-tailed independent samples *t* test was conducted to examine whether the difference in mean emotional sentiment between the r/LGBTeens and r/Relationships posts were statistically significant. Prior to the main analyses, the assumption of homogeneity of variance was checked; the results of a Levene test for r/Relationships and r/LGBTeens emotional tone was significant based on α=.05 (*F*_1,1403967_=37821.73, *P*<.001), indicating the assumption was violated. The result of the 2-tailed independent samples t-test suggested the mean emotional sentiment of r/LGBTeens and r/Relationships posts were significantly different based on α=.05 (*t*_42204.64=_77.99, *P*<.001). Since the assumption of assumption of homogeneity of variance was violated, a Mann-Whitney *U* Test, which does not have any distributional assumptions, was conducted to supplement the *t* test results. The result of the 2-tailed Mann-Whitney *U* test examining differences between r/LGBTeens and r/Relationships posts was significant based on α=.05 (*U*=4301049386.5, *z*=−Inf, *P*<.001), suggesting that the distribution of emotional tone in r/LGBTeens posts was significantly different than that in r/Relationships.

Additionally, a 2-tailed independent samples *t* test was conducted to compare the mean emotional sentiment of r/LGBTeens and r/Teenagers posts. The results of the Levene test comparing r/teenagers and r/LGBTeens posts also indicated the variances of tone for r/LGBTeens and r/Relationships posts were unlikely to be equal based on α=.05 (*F*_1,232265_=37821.73, *P*<.001). The results of the 2-tailed independent sample *t* test suggested the mean emotional sentiment of r/LGBTeens and r/teenagers posts were also significantly different, based on α=.05 (*t*_41019.96_=27.86, *P*<.001). The homogeneity of variance violation necessitated a Mann-Whitney *U* Test to supplement the *t* test results; results were significant based on α=.05 (*U*=28602717011.5, *z*=−27.23, *P*<.001), suggesting their distributions were significantly different.

Findings revealed that the emotional sentiment of r/LGBTeens posts (mean 42.34, SD 34.68) was significantly greater than those of r/Teenagers posts (mean 37.4, SD 32.95) and r/Relationships posts (mean 28.38, SD 15.57).

We found a decrease in emotional tone in late January in both r/Teenagers and r/LGBTeens posts when many schools closed temporarily (Figure 5). This downturn was followed by a second decrease in r/LGBTeens and r/Teenagers tone when public schools announced that closures would last through 2020. Then, there was a sharp rise in emotional tone during the Black Lives Matter protests and simultaneous Pride celebrations for r/LGBTeens posts but not for r/Teenagers posts. After Pride celebrations and Black Lives Matter protests in June, the trend for r/Teenagers posts seemed to stabilize. However, we saw a tone decrease in r/LGBTeens posts, representing more negatively valenced posts, which persisted until a second prominent increase in early November 2020. This November upsurge may be explained by the election of President Biden, who has a pro-LGBTQ+ agenda in contrast to his predecessor [35,37,47].

Furthermore, we ran a point biserial correlation analysis to assess whether negative emotion changed as US social distancing orders were relaxed and vaccine distribution increased in January 1, 2021 [50]. LIWC positive emotion calculates the frequency of positive emotion lexicon words (eg, “happy” or “thankful”) relative to the total number of words in a post. To supplement our results, we likewise assessed whether positive emotion changed as US social distancing orders were gradually lifted. While there were no significant differences in anger and sadness, results showed a significant increase in anxiety after social distance orders were reduced in January of 2021 (*r*=0.01, *P*=.04, 95% CI 0 to 0.02). Similarly, we found a decrease in positive emotion after social distancing orders began to be lifted (*r*=−0.01, *P*=.04, 95% CI −0.02 to 0). This decrease in positive emotion and increase in anxiety may be because—regardless of the vaccine becoming available on December 10, 2020 and reduced social distancing orders—youth were not immediately impacted (eg, the vaccine was not approved for youth ages 12 to 16 years until May of 2021 [51]). We would expect that this trend might flatten out in Fall 2021—when students begin to return to normalcy (Multimedia Appendix 6).

### Conversation Patterns That Manifest From Heightened Anxiety

**Figure 6:**
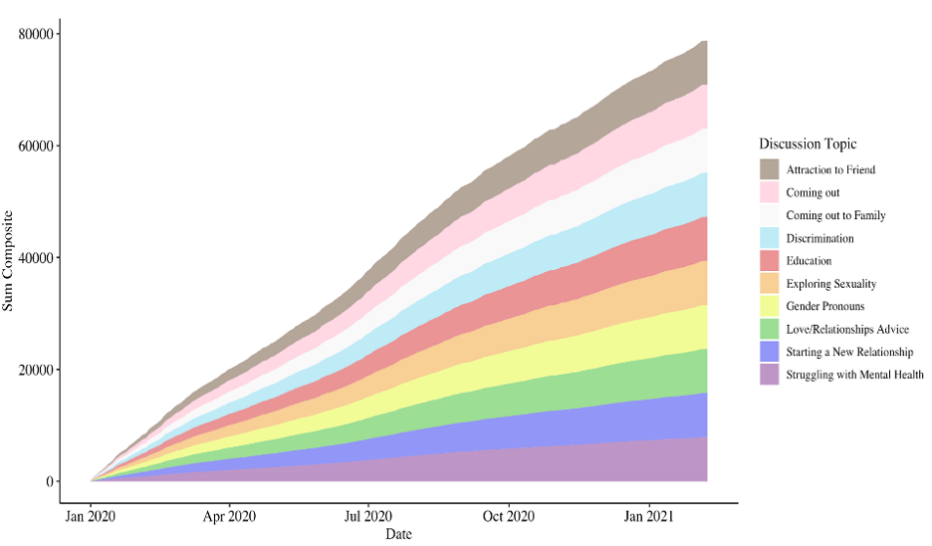
Streamgraph of topic percent contribution to the corpus of anxious r/LGBTeens posts over time. Each color represents a discussion topic across the corpus of anxious r/LGBTeens posts. This streamgraph shows the composite of topics overall (graph envelope) and the relative importance of the topic to posts over time (topic color stream width).

Because the distribution of anxiety before widespread social distancing orders was meaningfully different from the distribution after lockdown (*U*=125657033, *z*=−6.20, *P*<.001), we used a topic model to extrapolate the specific conversation topics co-occurring with LGBTQ+ teen anxiety. Findings revealed the following conversation topics among the LGBTQ+ youth during the pandemic accompany sharp increases in anxiety: attraction to a friend, coming out, coming out to family, discrimination, education, exploring sexuality, gender pronouns, love/relationship advice, starting a new relationship, and struggling with mental health. There was no meaningful change in the anxiety-provoking topics discussed over time (Multimedia Appendix 7); however, the frequency of such discussions increased (Figure 6).

## Discussion

We employed natural language processing to investigate the emotional trends in LGBTQ+ teens’ anonymous online conversations during the COVID-19 pandemic. Results revealed that the overall emotional tone of posts sharply decreased during the 2020-2021 COVID-19 crisis, relative to prior years—revealing this emotional trend was specific to the COVID-19 crisis. Findings reveal that the emotional trajectory of LGBTQ+ youth fluctuated more drastically in response to impactful events during the COVID-19 crisis (eg, widespread school closures and Black Lives Matter protests) compared to the emotional trajectory of more neutral subreddit spaces [37,39,40].

Findings revealed that the trajectory of LGBTQ+ teens’ overall emotional tone (positive vs negative) to be more affected by lifestyle stressors during the COVID-19 crisis than the general population of r/Teenage users. Results are consistent with those from previous research indicating that LGBTQ+ youth are disproportionately vulnerable to adverse mental health outcomes relative to their straight, cisgender peers [4-6].

While this study did not find pre and postlockdown differences in LGBTQ+ youth anger and sadness, results revealed that anxiety increased after government-mandated social distancing measures. In addition, further analysis revealed a list of 10 anxiety-provoking topics discussed during the pandemic: attraction to a friend, coming out, coming out to family, discrimination, education, exploring sexuality, gender pronouns, love/relationship advice, starting a new relationship, and struggling with mental health. These conversation topics were anxiety-provoking for LGBTQ+ youth both before and during the pandemic. However, the increase in the frequency of these conversations coincided with the emergence of lifestyle disruptors related to the pandemic, reflecting LGBTQ+ teens’ increased reliance on anonymous discussion forums as outlets for discussing lifestyle stressors during COVID-19 lifestyle disruptions (eg, school closures).

Findings revealing LGBTQ+ teens’ increased reliance on an anonymous forum as a discussion outlet during the COVID-19 outlet were consistent with those from previous studies showing that individuals are likely to turn to social media in times of crisis to seek psychological support and build community resilience [34]. Furthermore, the results of this study are in line with those of existing studies demonstrating the importance of online support to LGBTQ+ youth while coping with the challenges of a global pandemic [5].

This study also shed light on the specific sources of anxiety for LGBTQ+ youth during the COVID-19 pandemic. Research has revealed links between LGBTQ+ youth anxiety disorders and self-harm and suicidal behavior—in part due to stigma and discrimination [6]. Additionally, research has shown that elevated anxiety levels can weaken individuals’ immune systems, make them more vulnerable to certain illnesses, and elevate their risk of death from cardiovascular complications [52]. Clinical practices show that identifying sources of anxiety is an essential step in helping teenagers develop coping mechanisms [53]. By identifying sources of anxiety in LGBTQ+ youth, this study may help mental health professionals design effective strategies to address anxiety in that population.

Additionally, this study’s findings suggest that mental health professionals should consider anonymous online supplements or alternatives to in-person treatment of LGBTQ+ youth anxiety, especially during school closures. Despite mental health professionals’ adaptation to web-based counseling, LGBTQ+ youth report that treatment cost and parental consent are barriers to accessing mental health resources outside of school [10,11]. Findings accentuate the need to develop web-based programming to address the needs of LGBTQ+ youth in times of crisis and were consistent with those of Paceley et al [54]. Research shows that youth whose social environments support their LGBTQ+ identities are less likely to have suicidal thoughts or attempt suicide, compared to those who live in nonsupportive social environments [55]. Furthermore, being connected to the LGBTQ+ community may provide a buffer to suicidal thoughts [56]. Amid widespread social distancing orders, LGBTQ+ youth may have turned to web-based resources as a way of connecting with the LGBTQ+ community [57]. For instance, there was an upsurge of r/LGBTeens positive emotion during the rise of the Black Lives Matter protests, which may be explained by concurrent Gay Pride celebration unity, where many Pride celebrations stood in solidarity with Black Lives Matter to support marginalized populations [38-40]. The upsurge of positive emotion during Gay Pride was followed by another upsurge when President Biden won the US presidential election (Multimedia Appendix 8). Future work should consider how LGBTQ+ youth well-being may benefit from identity-affirming online spaces to celebrate their individual and collective achievements [34].

Although this study provides valuable insight into LGBTQ+ youth mental health during the COVID-19 pandemic, the study had some limitations. First, using computerized coding tools such as LIWC does not allow for sophisticated coding that could be achieved with human coders. Previous studies using LIWC have found that LIWC may overidentify emotional expression [58]; thus, LIWC may have captured extraneous sentiment. Second, research shows that observational studies may lead to faulty findings due to confounding factors. Although scholars admit that it is not possible to identify all confounders in practice [59], we expect that online expressions will vary between individuals according to their level of exposure to mental health risk factors, such as institutionalized and interpersonal discrimination and the lack of parental support [6,52]. Although these factors are consequential, we cannot test them using an observational study. To reduce bias and limitations through methodological triangulation, future research should marry observational work on LGBTQ+ youth with surveys and experimental data. Surveying LGBTQ+ youth may also help to identify the specific sources of emotional distress (eg, negative emotion resulting from the elimination of LGBTQ+ nondiscrimination protections vs negative emotion related to COVID-19 isolation).

The COVID-19 crisis has caused a concurrent mental health pandemic, and LGBTQ+ youth are especially vulnerable to adverse mental health outcomes [4-7]. Results reveal that online microcommunities serve as a viable space for LGBTQ+ youth to express their emotions about lifestyle stressors and navigate their stigmatized identities while simultaneously building community resilience [5,34]. Because LGBTQ+ self-disclosure is helpful for mental health [13,14], future work should explore the potential of web-based platforms in identifying the sources of LGBTQ+ youth emotional distress and developing safe online spaces to offer support to this vulnerable population.

## Appendix

Multimedia Appendix 1 Polynomial regression of Linguistic Inquiry Word Count anxiety levels in r/LGBTeens posts from January 1, 2015 through January 31, 2021.

Multimedia Appendix 2 Perplexity metric.

Multimedia Appendix 3 Latent Dirichlet allocation results.

Multimedia Appendix 4 Intertopic distance.

Multimedia Appendix 5 Topic model data processing.

Multimedia Appendix 6 Point biserial correlation analysis.

Multimedia Appendix 7 Anxiety-related topics over time.

Multimedia Appendix 8 Histogram of the percentage of positive sentiment in r/LGBTeens posts over time and average level of positive emotion in positive r/LGBTeens posts over time.

## Abbreviations

LDA: latent Dirichlet allocation
LGBTQ+: Lesbian, Gay, Bisexual, Transgender, Queer/Questioning, and Others
LIWC: Linguistic Inquiry and Word Count program
