# Supplementary material for: Natural Language Processing Insight into LGBTQ+ Youth Mental Health During the COVID-19 Pandemic: Longitudinal Content Analysis of Anxiety-Provoking Topics and Trends in Emotion in LGBTeens Microcommunity Subreddit
Source: JMIR Public Health Surveill. 2021 Aug 17;7(8):e29029. doi: 10.2196/29029 (PMC8372845; doi:10.2196/29029)
Supplement: Multimedia Appendix 1 [file publichealth_v7i8e29029_app1.docx]

**Multimedia Appendix 1.**

Polynomial regression of average Linguistic Inquiry Word Count (LIWC) Anxiety levels of anxious r/LGBTeens posts from January 1, 2015 through January 31, 2021; the data showed an unprecedented upsurge in anxiety between January 1, 2020 and January 1, 2021.


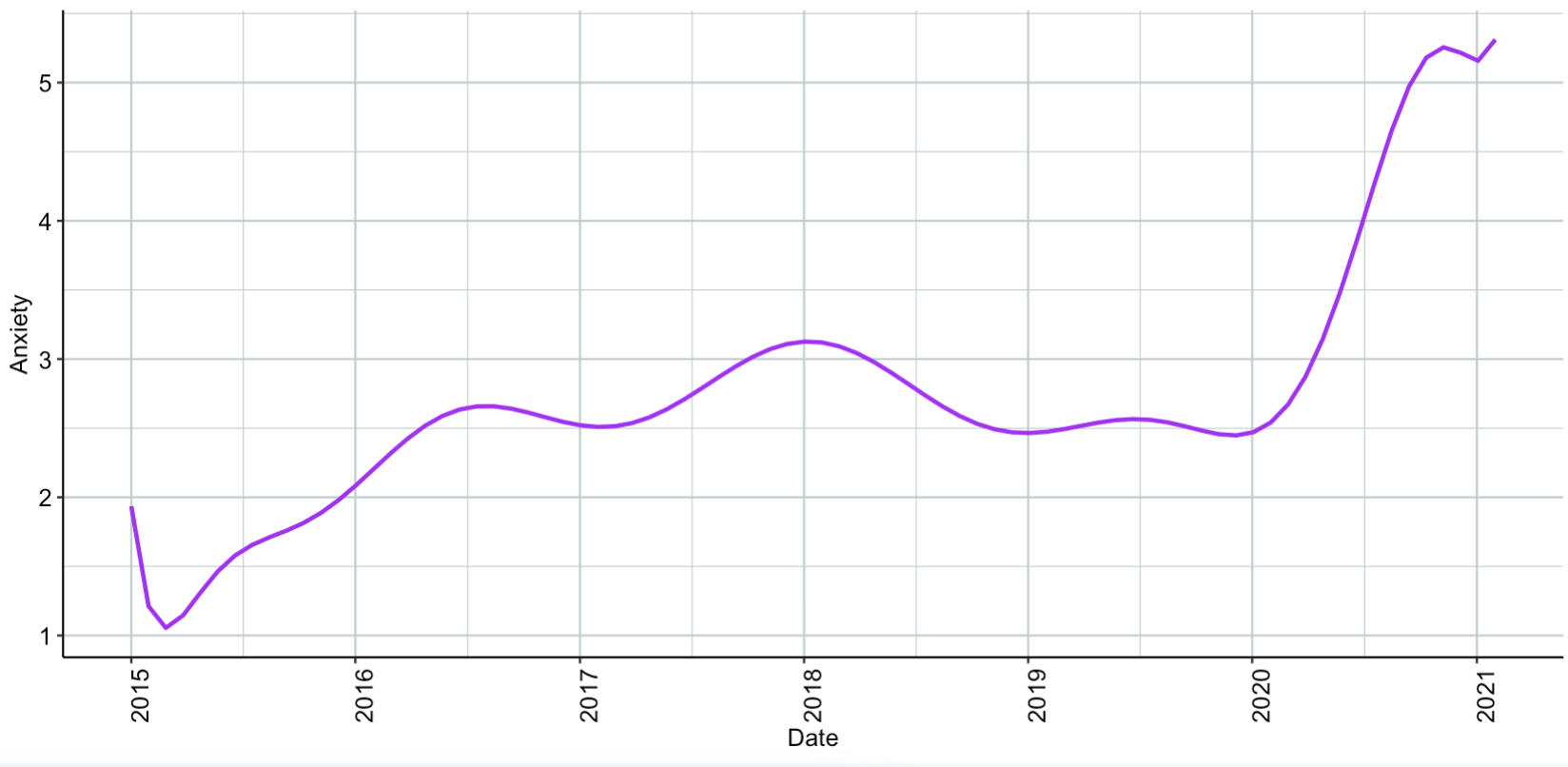


**Figure A1.** *Polynomial regression line representing average LIWC Anxiety levels of anxious r/LGBTeens posts from January 1, 2015 through January 31, 2021*
