# Supplementary material for: Natural Language Processing Insight into LGBTQ+ Youth Mental Health During the COVID-19 Pandemic: Longitudinal Content Analysis of Anxiety-Provoking Topics and Trends in Emotion in LGBTeens Microcommunity Subreddit
Source: JMIR Public Health Surveill. 2021 Aug 17;7(8):e29029. doi: 10.2196/29029 (PMC8372845; doi:10.2196/29029)
Supplement: Multimedia Appendix 2 [file publichealth_v7i8e29029_app2.docx]

**Multimedia Appendix 2.**

The perplexity metric (the normalized log-likelihood of the model finding a previously unseen term) was used to identify the optimal (k) number of topics for the model. Lower perplexity values suggest greater model accuracy.
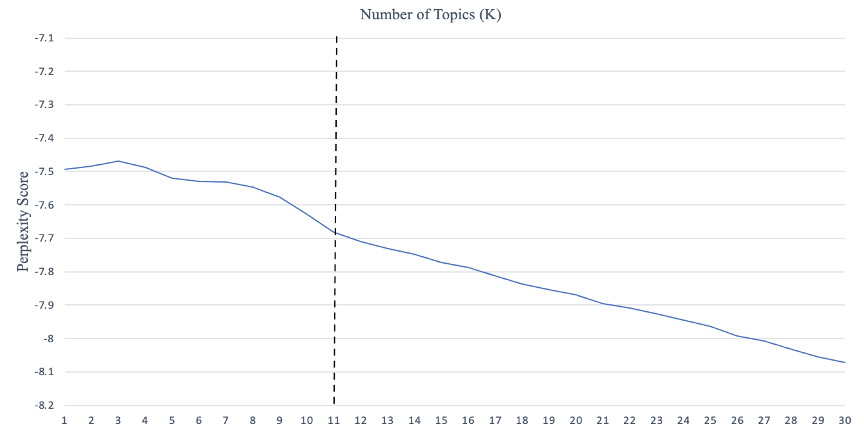


**Figure A2.** *Perplexity scores for k=1-30 topics. The model with k=11 topics yielded the lowest perplexity before the trend flattens out.*
