# Supplementary material for: Natural Language Processing Insight into LGBTQ+ Youth Mental Health During the COVID-19 Pandemic: Longitudinal Content Analysis of Anxiety-Provoking Topics and Trends in Emotion in LGBTeens Microcommunity Subreddit
Source: JMIR Public Health Surveill. 2021 Aug 17;7(8):e29029. doi: 10.2196/29029 (PMC8372845; doi:10.2196/29029)
Supplement: Multimedia Appendix 3 [file publichealth_v7i8e29029_app3.docx]

**Multimedia Appendix 3.**

The Latent Dirichlet Allocation (Latent Dirichlet Allocation (LDA) model generated a list of the top 10 terms contributing to each topic and their beta values (β). Because coders deemed topic 4 incoherent, we omitted it. These topics and terms are visualized in Figure A3.

Table A1. Most Influential Term for each r/LGBTeen Topic

| No. | Label | Most influential terms (β) |
| --- | --- | --- |
| 1 | Education | go (0.033), school (0.020), get (0.018), day (0.015), year (0.014), month (0.012), start (0.010), time (0.010), week (0.010), last (0.009) |
| 2 | Love/Relationship Advice | feel (0.088), like (0.048), really (0.029), want (0.025), know (0.023), would (0.020), think (0.020), feel like (0.018), even (0.016), relationship (0.013) |
| 3 | Starting a Relationship | like (0.021), talk (0.019), girl (0.018), one (0.016), date (0.015), get (0.014), boyfriend (0.013), first (0.012), guy (0.012), know (0.011 |
| 4 | Incoherent | get (0.015), love (0.014), life (0.013), see (0.012), look (0.011), thing (0.009), people (0.008), much (0.008), want (0.008), watch (0.008) |
| 5 | Coming Out | tell (0.075), say (0.052), know (0.049), want (0.038), friend (0.030), gay (0.029), people (0.029), ask (0.021), go (0.016), talk (0.011) |
| 6 | Discrimination | like (0.034), say (0.032), make (0.025), gay (0.025), people (0.018), get (0.016), even (0.012), bad (0.011), shit (0.009), fuck (0.009) |
| 7 | Exploring Sexuality | girl (0.049), guy (0.031), think (0.027), sexuality (0.027), like (0.026), bisexual (0.025), know (0.016), man (0.014), lesbian (0.014), find (0.014) |
| 8 | Attraction to Friends | crush (0.072), friend (0.067), really (0.047), like (0.038), good (0.023), know (0.021), kinda (0.021), think (0.017), also (0.016), guy (0.014) |
| 9 | Gender Pronouns | gender (0.040), like (0.019), use (0.017), name (0.016), want (0.014), non (0.014), know (0.014), pronoun (0.014), think (0.013), binary (0.012) |
| 10 | Coming out to Family | come (0.166), parent (0.045), family (0.039), mom (0.038), accept (0.025), dad (0.024), scared (0.024), know (0.017), scare (0.017), homophobic (0.014) |
| 11 | Struggling with Mental Health | help (0.124), confused (0.104), advice (0.078), need (0.072), please (0.044), struggle (0.037), panic (0.018), anxiety (0.017), anyone (0.016), thank (0.013) |
