# Supplementary material for: Natural Language Processing Insight into LGBTQ+ Youth Mental Health During the COVID-19 Pandemic: Longitudinal Content Analysis of Anxiety-Provoking Topics and Trends in Emotion in LGBTeens Microcommunity Subreddit
Source: JMIR Public Health Surveill. 2021 Aug 17;7(8):e29029. doi: 10.2196/29029 (PMC8372845; doi:10.2196/29029)
Supplement: Multimedia Appendix 4 [file publichealth_v7i8e29029_app4.docx]

**Multimedia Appendix 4**

We visualized the inter-topic distance with the dynamic PyLDAvis python package, which enables human coders to view a 3-dimensional LDA inter-topic map in 2 dimensions (Figure A3).


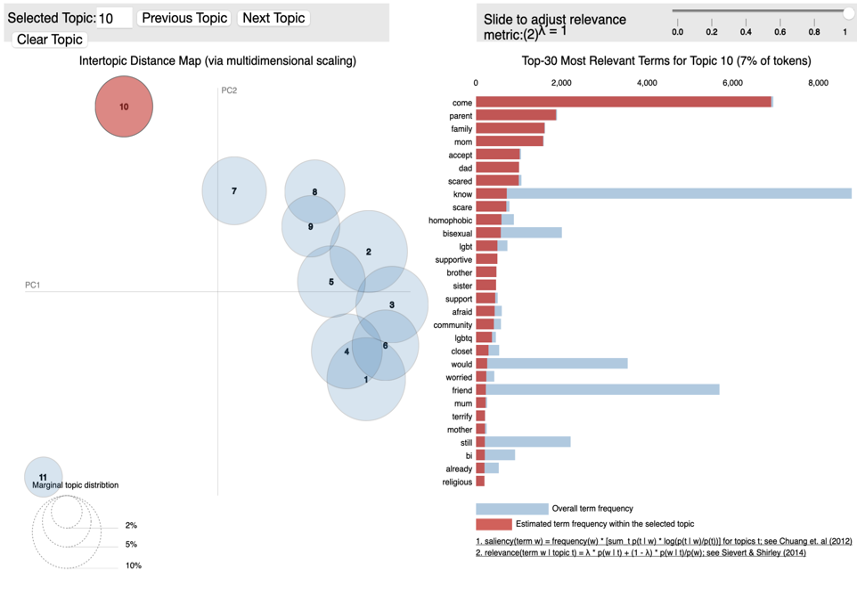


Figure A3. *Inter-topic distance map created with PyLDAvis*
