# Supplementary material for: Natural Language Processing Insight into LGBTQ+ Youth Mental Health During the COVID-19 Pandemic: Longitudinal Content Analysis of Anxiety-Provoking Topics and Trends in Emotion in LGBTeens Microcommunity Subreddit
Source: JMIR Public Health Surveill. 2021 Aug 17;7(8):e29029. doi: 10.2196/29029 (PMC8372845; doi:10.2196/29029)
Supplement: Multimedia Appendix 5 [file publichealth_v7i8e29029_app5.docx]

**Multimedia Appendix 5.**

Visual representation of data processing using the LDA topic model.


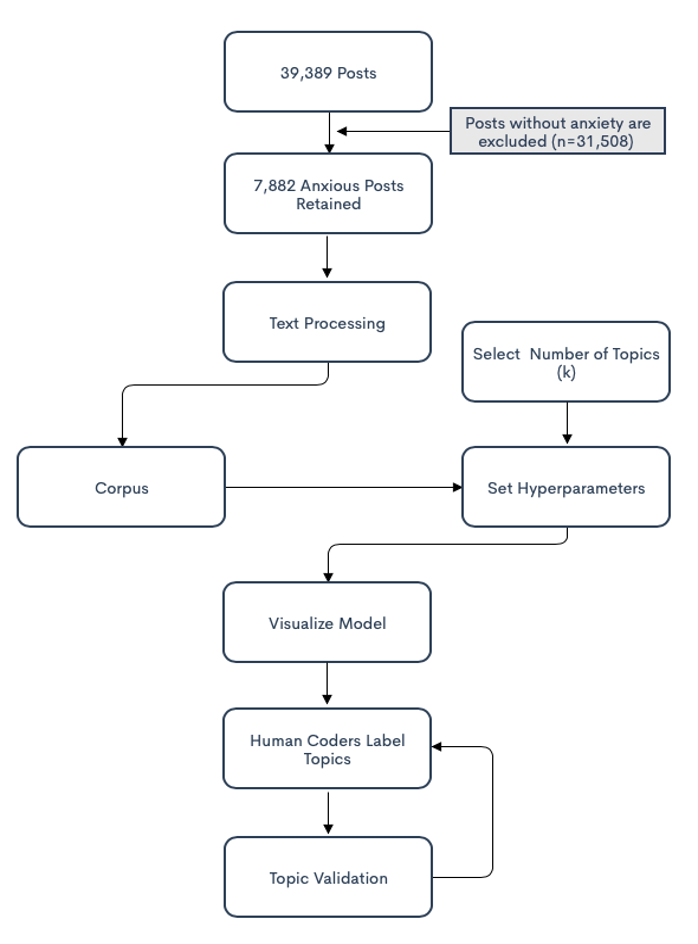


Figure A4. *LDA Topic Model processing steps.*
