# Supplementary material for: Natural Language Processing Insight into LGBTQ+ Youth Mental Health During the COVID-19 Pandemic: Longitudinal Content Analysis of Anxiety-Provoking Topics and Trends in Emotion in LGBTeens Microcommunity Subreddit
Source: JMIR Public Health Surveill. 2021 Aug 17;7(8):e29029. doi: 10.2196/29029 (PMC8372845; doi:10.2196/29029)
Supplement: Multimedia Appendix 6 [file publichealth_v7i8e29029_app6.docx]

**Multimedia Appendix 6.**

We ran a point biserial correlation analysis to assess whether emotion changed as U.S. social distancing orders were lifted (in January 2021). Holm corrections were applied to *p*-values to adjust for multiple comparisons based on an alpha of 0.05. While there were no significant differences in anger and sadness, results showed a significant positive correlation between anxiety and the gradual reduction of social distancing orders in January of 2021 (*r*_pb_ = 0.01, *p* = .038, 95% CI [0.00, 0.02]). Similarly, there was a significant negative correlation between positive emotion and the reduction of social distancing orders (*r*_pb_ = -0.01, *p* = .036, 95% CI [-0.02, -0.00]). The results are presented in Table A2.

Table A2. Point Biserial Correlation Analysis

| Combination | *r*_pb_ | 95% CI | *p* |
| --- | --- | --- | --- |
| Reduced Social Distancing Mandates-anxiety | 0.01 | [0.00, 0.02] | .038 |
| Reduced Social Distancing Mandates-sadness | 0.01 | [0.00, 0.02] | .094 |
| Reduced Social Distancing Mandates-anger | 0.00 | [-0.01, 0.01] | .952 |
| Reduced Social Distancing Mandates-positive emotion | -0.01 | [-0.02, -0.00] | .036 |
