# Supplementary material for: Natural Language Processing Insight into LGBTQ+ Youth Mental Health During the COVID-19 Pandemic: Longitudinal Content Analysis of Anxiety-Provoking Topics and Trends in Emotion in LGBTeens Microcommunity Subreddit
Source: JMIR Public Health Surveill. 2021 Aug 17;7(8):e29029. doi: 10.2196/29029 (PMC8372845; doi:10.2196/29029)
Supplement: Multimedia Appendix 7 [file publichealth_v7i8e29029_app7.docx]

**Multimedia Appendix 7.**

We did not find meaningful differences in the relative contribution of specific anxious topics over time (Figure A5).


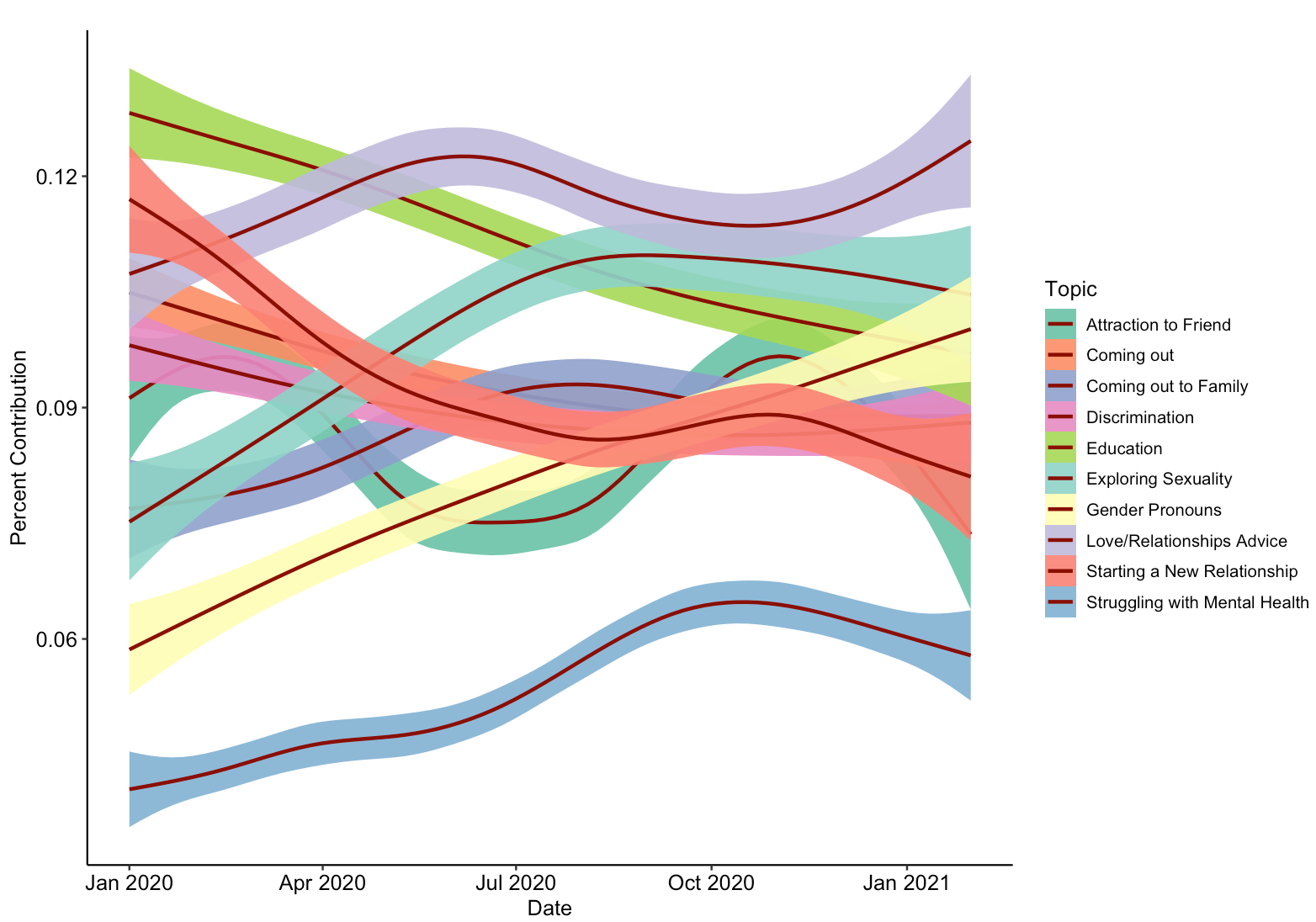


Figure A5. *Polynomial regression lines showing the average popularity of each topic over time.*
